# Extramedullary Disease in Acute Promyelocytic Leukemia: Two-In-One Disease

**DOI:** 10.4084/MJHID.2011.066

**Published:** 2011-12-21

**Authors:** Francesco Albano, Giorgina Specchia

**Affiliations:** Ematologia con Trapianto, Università degli Studi di Bari “Aldo Moro“

## Abstract

In acute promyelocytic leukemia (APL), extramedullary disease (EMD) is particularly rare and shows special clinical and biological features. It is estimated that about 3–5% of APL patients will suffer extramedullary relapse. The most common site of EMD in APL is the central nervous system (CNS). At present, there are still many issues of EMD in APL needing further clarification, including pathogenesis, risk factors, prognosis and treatment. A better understanding of the biological mechanisms underlying EMD is important to be able to devise more effective CNS prophylaxis and induction-consolidation therapeutic strategies.

## Introduction

Acute leukemia may present in a variety of extramedullary tissues with or without bone marrow disease. Extramedullary involvement by acute leukemia is a relatively rare but clinically significant phenomenon that often poses diagnostic and therapeutic dilemmas. Myeloid sarcoma and leukemia cutis are two well-known EM manifestations. Extramedullary disease (EMD) in acute promyelocytic leukemia (APL) is particularly rare and shows special clinical and biological features.

## How common is EMD in APL?

The combination of all-trans retinoic acid (ATRA) and anthracycline-based chemotherapy, together with maintenance treatment, has improved the outcome of APL. In fact, approximately 90% of patients with newly diagnosed APL achieve complete remission (CR).^1^–^2^ and it is estimated that 70–80% of these patients will remain in remission.^1^,^3^ However, approximately 20–30% of patients will eventually relapse.^2^ EMD is a rare complication in APL: it is estimated that about 3–5% of patients will suffer extramedullary relapse.^4^–^6^ However, since the introduction of ATRA in the treatment of patients with APL, EMD disease has been increasingly reported; in fact, in the literature fewer than 25 well-documented cases had been described before 1995.^7^ This is most likely in part due to the following reasons:

- APL patients may develop EMD more frequently because they are achieving longer survival times thanks to improved treatment regimens.
- It is possible that the drugs employed in the induction regimens (ATRA, anthracycline and arsenic trioxide) do not reach therapeutic concentrations at the anatomical sites where EMD eventually develops.
- It is also possible that ATRA therapy might contribute to extramedullary relapses by modulating and upregulating the expression of adhesion molecules on leukemic cells.

EMD commonly occurs within 1 year of achieving CR, but it can appear at any time during the disease course and can be isolated or can precede systemic relapse.^2^,^6^ As to cases of EMD at APL presentation, although a few anecdotal reports have been made this observation is very uncommon.^8^

## What are the most frequent anatomical sites of EMD in APL?

**Figure 1:**
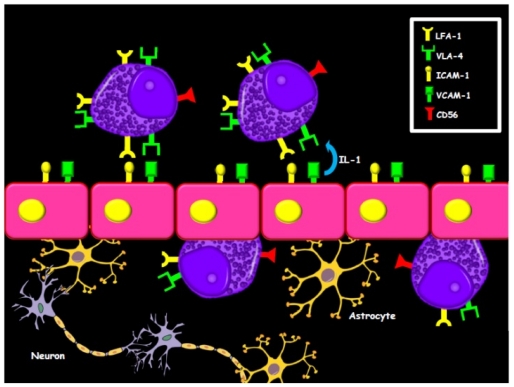
ATRA-driven differentiation of APL cells is associated with the upregulation of cellular adhesion molecules like LFA-1 and VLA-4. The mechanism of APL blasts adhesion to the endothelium may be further increased by interleukin-1, an effect which may be mediated via an increased expression of ICAM-1 and VCAM-1 on the CNS endothelium. Since both LFA-1 and VLA-4 are upregulated in APL blasts treated with retinoids, it is reasonable to suppose that the upregulation of these adhesion molecules might promote passage across the BBB of ATRA-treated APL cells, thereby creating the conditions for a subsequent CNS relapse. Moreover, CD56 expression on APL cells may also foster CNS relapse.

The most frequent site of EMD in APL patients is the central nervous system (CNS) and at least 10% of hematologic relapses are accompanied by CNS involvement.^9^ CNS relapse appears in around 1% of APL patients and may occur despite hematological remission.^6^,^10^–^12^ The skin is the second most common site of EMD.^2^ The increased frequency of EMD especially in these two sites could be explained by some biological effects of ATRA induction treatment. In fact, ATRA-driven differentiation of APL cells is associated with a significant upregulation of cellular adhesion molecules expressed on the cell surface, like LFA-1 and VLA-4.^13^ The mechanism of APL blasts adhesion to the endothelium may be further increased by interleukin-1, via an effect which may be mediated through an increased expression of ICAM-1 and VCAM-1 on the endothelial cell surface.^14^ These surface proteins have both been demonstrated on the CNS endothelium and have been implicated in the migration processes of leukocytes across the blood–brain barrier (BBB), through interactions with LFA-1 and VLA-4, respectively.^9^ Since both LFA-1 and VLA-4 are upregulated in APL blasts treated with retinoids, it is reasonable to suppose that the upregulation of these adhesion molecules may promote passage across the BBB of ATRA-treated APL cells, thereby creating the conditions for a subsequent CNS relapse (Figure 1). Moreover, ATRA also stimulates keratinocytes to proliferate and upregulate their expression of ICAMs.^9^ It has been suggested that the migration of leukemic cells into the skin and other tissues during ATRA induction treatment may leave a reservoir of viable leukemic cells in these sites, that eventually may proliferate and cause EMD. These biological events could account for the clinical observation of a preferential skin localization of APL cells relapsing after ATRA treatment. Moreover, a high frequency of EMD in APL may also be related to the ATRA-induced upregulation of G-CSF receptors in APL cells, making them more sensitive to endogenous or exogenous G-CSF effects.^15^ Other described sites of EMD in APL include: the testes, sites of vascular access, external ear and auditory canal, lung, pleura, heart, lymph nodes, mediastinum, thymus, spine, breast, pelvis, mandible and gingiva, bowel. Since in patients affected by ATRA syndrome APL cells infiltrate multiple tissues and organs, it has been hypothesized that ATRA could promote the migration of differentiating blasts into several tissues, constituting a reservoir of viable leukemic cells. These cells could later proliferate and result in an extramedullary recurrence.^16^–^17^ However, the issue as to whether ATRA promotes EMD in APL is still highly controversial, since several studies have reached different conclusions.^5^–^7^,^18^–^19^

## Are there risk factors for an EMD onset in APL?

Several factors have been associated with a higher risk of extramedullary relapse such as younger age (<45 years), a high WBC count at diagnosis, microgranular morphology, expression of CD2 and/or CD56, PML-RARα bcr3 isoform expression, ATRA syndrome, monotherapy regimens, and the use of therapy schedules that exclude cytarabine.^2^,^4^,^12^,^20^–^21^ Moreover, two recent studies^4^,^22^ reported a significantly higher incidence of CNS involvement in patients with an initial WBC of more than 10 ×10^9^/L. In addition to hyperleukocytosis, the PETHEMA study also identified a previous CNS hemorrhage during induction as an independent risk factor for CNS relapse.^22^ It has recently been demonstrated that CD56+ APL has a greater risk of extramedullary relapse.^21^ The higher frequency of coexpression of stem cell (CD117) and NK-cell antigens (CD2, CD7) in CD56+ APL cells suggests that in some of these cases APL might have arisen in progenitors that did not undergo lineage restriction.^23^ Therefore, it is possible that CD56+ APL may emerge from a more immature, undifferentiated and pluripotent leukemic stem cell that is less sensitive to the combination of ATRA and anthracyclines. This could explain the higher frequency of extramedullary relapse in these cases.^21^,^24^–^25^

## What is the prognosis of EMD in APL? Which is the best therapy?

Because of the rarity of the disease, the prognosis of patients with EMD in APL is still unclear. The GIMEMA study^6^ reported that the outcome was similar to that of patients who experienced isolated bone marrow relapse, whereas in the joint study by the PETHEMA and the European APL groups^4^ it was found that patients with an extramedullary relapse had a poorer outcome. EMD can occur in isolation or associated with bone marrow involvement as a first relapse, but also after one or more hematologic relapses. The molecular status in the peripheral blood/bone marrow did not seem to predict the possibility of EMD relapse.^5^–^6^ Management of relapse in the CNS and other extramedullary sites in APL patients is a challenging issue on which there is a strong need for further data. The optimal management of APL patients in different situations has not been critically assessed.^26^ Because the majority of CNS relapses occurs in APL patients with hyperleukocytosis,^4^ CNS prophylaxis for patients in this particular high-risk setting may be appropriate.^26^ In these cases CNS prophylaxis should be performed after the achievement of CR because lumbar puncture at presentation and during induction is extremely hazardous. However, the benefit of this kind of strategy has not yet been clearly established.

The role of ATRA and arsenic trioxide in the therapeutic management of CNS relapse is still unclear because it is not known whether these drugs cross the BBB; nevertheless, some authors have reported responses to these agents in patients with meningeal disease.^27^–^28^ This may be due to the EMD disrupting the BBB. Arsenic trioxide has also been reported to cross the BBB and may be useful as a therapeutic agent to control CNS relapse.^29^ On the other hand, some reports have confirmed that although arsenic crosses the BBB when administered intravenously, the concentration in CSF is probably not sufficient to treat meningeal leukemia.^30^–^31^ Recently, as induction treatment of CNS relapse, the European LeukemiaNet recommendations^26^ proposed a schedule of weekly triple intrathecal therapy (ITT) with methotrexate, hydrocortisone, and cytarabine until complete clearance of blasts in the cerebrospinal fluid (CSF), followed by 6 to 10 more spaced-out ITT treatments as consolidation. In these cases systemic treatment should also be given because CNS disease is almost invariably associated with hematologic or molecular relapse in the marrow. Chemotherapy regimens with high CNS penetrance, such as high-dose cytarabine, have been used in this situation. In patients responding to treatment, allogeneic or autologous transplant is then recommended as consolidation treatment, together with craniospinal irradiation. It was demonstrated that cytarabine during consolidation treatment significantly reduced the relapse rate in high-risk APL patients.^32^–^33^ Because of the limited numbers of EMD events reported in these studies, it is very hard to draw firm conclusions regarding the best schedule of cytarabine to use in the consolidation regimen to prevent the EMD in APL. In cases of promyelocytic sarcoma, wherever it is localized, radiation and intensive systemic therapy might be considered. Recently, successful treatment of relapsed and refractory EMD with Tamibarotene,^34^ a synthetic retinoid approved in Japan for use in relapsed/refractory APL, has been reported.^35^ Tamibarotene is 10 times more potent than ATRA as an inducer of HL-60 and NB-4 leukemia cell lines differentiation. While tamibarotene has displayed a significant activity in bone marrow-relapsed APL, its efficacy in EMD needs to be confirmed in further studies.

## Conclusions

At present, there are still many open issues on EMD in APL patients. However, some aspects are becoming clearer. An improved understanding of the biological mechanisms that underlie EMD should allow us to devise more effective prophylaxis and induction therapeutic strategies against this severe clinical presentation.
